# GLUT4 and UBC9 Protein Expression Is Reduced in Muscle from Type 2 Diabetic Patients with Severe Insulin Resistance

**DOI:** 10.1371/journal.pone.0027854

**Published:** 2011-11-16

**Authors:** Ulla Kampmann, Britt Christensen, Thomas Svava Nielsen, Steen Bønløkke Pedersen, Lotte Ørskov, Sten Lund, Niels Møller, Niels Jessen

**Affiliations:** 1 Department of Endocrinology and Internal Medicine, Aarhus University Hospital, Aarhus, Denmark; 2 Department of Internal Medicine, Silkeborg Regional Hospital, Silkeborg, Denmark; 3 Department of Clinical Pharmacology, Aarhus University Hospital, Aarhus, Denmark; University of Las Palmas de Gran Canaria, Spain

## Abstract

**Aims:**

Subgroups of patients with type 2 diabetes mellitus demand large insulin doses to maintain euglycemia. These patients are characterized by severe skeletal muscle insulin resistance and the underlying pathology remains unclear. The purpose of this study was to examine protein expression of the principal glucose transporter, GLUT4, and associated proteins in skeletal muscle from type 2 diabetic patients characterized by severe insulin resistance.

**Methods:**

Seven type 2 diabetic patients with severe insulin resistance (mean insulin dose 195 IU/day) were compared with seven age matched type 2 diabetic patients who did not require insulin treatment, and with an age matched healthy control group. Protein expression of GLUT4 and associated proteins was assessed in muscle and fat biopsies using standard western blotting techniques.

**Results:**

GLUT4 protein expression was significantly reduced by ∼30 pct in skeletal muscle tissue from severely insulin resistant type 2 diabetic subjects, compared with both healthy controls and type 2 diabetic subjects that did not require insulin treatment. In fat tissue, GLUT4 protein expression was reduced in both diabetic groups. In skeletal muscle, the reduced GLUT4 expression in severe insulin resistance was associated with decreased ubiquitin-conjugating enzyme 9 (UBC9) expression while expression of GLUT1, TBC1D1 and AS160 was not significantly different among type 2 diabetic patients and matched controls.

**Conclusions:**

Type 2 diabetic patients with severe insulin resistance have reduced expression of GLUT4 in skeletal muscle compared to patients treated with oral antidiabetic drugs alone. GLUT4 protein levels may therefore play a role in the pathology behind type 2 diabetes mellitus among subgroups of patients, and this may explain the heterogeneous response to insulin treatment. This new finding contributes to the understanding of the underlying mechanisms for the development of extreme insulin resistance.

## Introduction

Type 2 diabetic patients with extreme insulin resistance represent a major therapeutic challenge in terms of achieving glycaemic goals. Many patients require several hundred units of insulin daily, but despite large amounts of daily insulin, glycemic control remains poor, and it may be difficult to decide whether to increase insulin dosages further or regard patients as being non-compliant. It is unknown whether such patients have defects in the mechanisms that control insulin stimulated glucose uptake.

Skeletal muscle is the predominant tissue for insulin stimulated glucose disposal in humans [1]. Glucose enters the muscle cell primarily by facilitated diffusion, utilizing glucose transporter carrier proteins [2]. GLUT4 is the predominantly expressed glucose transporter isoform in muscle, whereas the GLUT1 isoform is expressed at much lower abundance [2]. In healthy subjects, whole body glucose disposal and GLUT4 expression correlate [1]. Glucose uptake through GLUT4 is regulated by insulin [2]. After binding to its receptor, insulin stimulates an intracellular signaling cascade that ultimately results in the phosphorylation of the Rab-GTPase-activating proteins AS160 (TBC1D4) and TBC1D1 [3]. When AS160 and TBC1D1 are phosphorylated on key residues, GLUT4 is released and translocates to the cell surface where it docks and fuses with the membrane [3]. After translocation to the cell surface, GLUT4 can either be recycled back to intracellular vesicles or be targeted for lysosomal degradation [4]. This process is regulated by ubiquitin-conjugating enzyme 9 (UBC9) which controls attachment of Small Ubiquitin-like Modifier (SUMO) proteins and thereby regulates degradation of GLUT4 in L6 muscle cells [5]. Similar observations have been made in 3T3-L1 adipocytes where overexpression of UBC9 promotes accumulation of GLUT4, whereas RNAi-mediated depletion of UBC9 causes a selective loss of GLUT4 [6].

Resistance to insulin in skeletal muscle and impaired insulin stimulated glucose uptake is a prominent feature of type 2 diabetes mellitus. This is associated with reduced insulin stimulated GLUT4 translocation demonstrated by labeling of GLUT4 on the cell surface [7], most likely caused by a reduced insulin signaling from the insulin receptor [8]. In adipose tissue, type 2 diabetes is associated with reduced GLUT4 expression [2], but in patients that maintain euglycemia on diet and treatment with oral antidiabetic drugs, the impaired translocation is not due to reduced expression of GLUT4 in skeletal muscle [2], [9]–[11]. However, type 2 diabetic patients who daily require high doses of insulin may have additional pathological defects in their muscles, and understanding these mechanisms is important for optimizing treatment. We therefore examined expression of GLUT4 and associated proteins in skeletal muscle from type 2 diabetic patients characterized by severe insulin resistance.

## Methods

The studies were conducted in accordance with the Helsinki Declaration and the study protocols were approved by the Ethical Committee of Central Denmark Region. Informed consent was obtained in writing from all subjects before participation.

Three groups were included: 1) type 2 diabetic patients with severe insulin resistance (T2DSI), defined as insulin requirements of more than 100 units/day, this group has been described previously [12], 2) a group of age matched type 2 diabetic patients treated with oral anti-diabetics or diet alone (T2DOAD), and 3) an age matched control group without concomitant disease. All participants had normal renal and hepatic function assessed by a blood test before they were included in the study. Oral anti-diabetic treatment was withdrawn two days before the study in both diabetic groups. The day before the study the patients in the T2DSI group had their usual insulin treatment replaced with short acting insulin (Actrapid; Novo Nordisk, Bagsværd, Denmark). All other treatments continued during the study. Average duration and type of daily physical activity was registered by the participants, and the data are presented as active hours per week. None of the participants were engaged in any type of strenuous physical activity. After an 8-h overnight fast, the subjects had blood samples drawn and a biopsy from the lateral vastus muscle was obtained under local anesthesia using a Bergström needle. The muscle biopsies were snap-frozen in liquid nitrogen and stored at −80°C until analyses were performed. One hour after the biopsy was taken the diabetic subjects underwent a 150 min hyperinsulinemic euglycemic clamp. In short, insulin (Actrapid) was infused at a rate of 5 ml/hour (1.5 mU*kg^−1^*min^−1^), and plasma glucose was clamped at 6 mM by infusion of a 20% glucose solution. Insulin sensitivity was estimated by the rate of glucose infusion required to maintain euglycemia during the insulin infusion and expressed as insulin stimulated glucose uptake (M-value). Body composition (Total Body Water (TBW)) was estimated by using single frequency bioelectrical impedance analysis (SF-BIA; BIA 101, RJL-Systems, MI, USA). TBW was calculated by using the equation TBW  = ρ×length^2^/Z [13].

### Blood samples and measurements

Plasma glucose was measured on a Beckman Glucoanalyzers (Beckman Instruments, USA). Plasma creatinine, lipids, and HbA_1c_ were determined by standard laboratory techniques. Insulin was analysed using TR-IFMA (AutoDELFIA, PerkinElmer, Finland) and C-peptide was measured by ELISA (DakoCytomation, United Kingdom).

### Western Blot analysis

Protein expression was assessed using standard western blotting techniques [14], [15]. In short, biopsies (∼30 mg) were homogenized in a buffer containing: 50 mM HEPES, 137 mM NaCl, 10 mM Na_4_P_2_O_7_, 10 mM NaF, 1 mM MgCl_2_, 1 mM CaCl_2_, 2 mM EDTA, 1% NP−40, 10% glycerol, 2 mM Na_3_VO_4_, 100 mM AEBSF [4-(2-aminoethyl) benzenesulfonyl fluoride, hydrocloride], pH 7.4. Protein expression was assessed using standard western blotting techniques. In short, protein was separated on a 4–12% Bis-Tris SDS gel (Criterion^TM^ XT, Bio-Rad, CA, USA), transferred to a PVDF membrane, and immunoblotted. β-actin (Abcam, #ab82227-50) was used as a control for equal loading. Antibodies against GLUT4 were generated as previously described [16] and commercially available antibodies against: AS160 (Cell Signalling, #2447), TBC1D1 (Abcam, #ab56191-100), UBC9 (Abcam, #ab75854), and GLUT1 (Millipore, #07-1401) were used. HRP-conjugated goat anti-rabbit IgG (#NA934, GE healthcare) was used as secondary antibody.

### Isolation of RNA

Skeletal muscle (20 mg) was homogenized in TriZol reagent (Gibco BRL, Life Technologies, Roskilde, Denmark). RNA was quantified by measuring absorbence at 260 nm and 280 nm and the integrity of the RNA was checked by visual inspection of the two ribosomal RNAs on an ethidium bromide stained agarose gel.

### Real-time RT-PCR for mRNA analysis

Reverse transcription was performed using random hexamer primers as described by the manufacturer (GeneAmp RNA PCR Kit from Perkin Elmer Cetus, Norwalk, CT). PCR-mastermix containing the specific primers and Taq DNA polymerase (HotStar Taq, Quiagen Inc. USA) was added. The following primers were designed using the primer analysis software Oligo version 6.64:

SOCS3:, CGG CCA CCT GGA CTC CTA TGA and GCC CTT TGC GCC CTT T

PTP1:; GCA CCC TAC GGC ATC GAA AG and GCA GCC TGG GCA CCT CGA AGA

GLUT4: GCA CCG CCA GGA CAT TGT TG and CCC CCT CAG CAG CGA GTG A

β2-microglobulin: AAT GTC GGA TGG ATG AAA CC and TCT CTC TTT CTG GCC TGG AG

Real time quantitation of target gene to β2-microglobulin mRNA was performed with a SYBR-Green real-time PCR assay using an ICycler from BioRad. The threshold cycle (Ct) was calculated, and the relative gene-expression was calculated essentially as described in the User Bulletin #2, 1997 from Perkin Elmer (Perkin Elmer Cetus, Norwalk, CT).

### Statistics

Results are expressed as mean±SE (parametric data) or median and range (non-parametric data). Statistical comparisons among groups were performed by One-Way ANOVA using SigmaPlot 11.0 (Systat Software inc. San Jose, CA, USA). LDL, HDL, TG, M-values, UBC9 expression, Real-time RT-PCR and activity levels were logarithmically transformed before applying parametric tests. *Post hoc* comparisons were performed with Holm-Sidak's test (parametric data) or Dunn's test (non-parametric data). Comparisons between two groups were assessed using a two-sample t-test. All data presented in tables have been re-transformed in order to make values immediately recognizable. P<0.05 was considered statistically significant.

## Results

Clinical characteristics of the participants are summarized in Table 1. On average, the T2DSI group received an insulin dose of 195 IU/day. Insulin treatment comprised either Novomix (n = 5), Lantus (n = 1), or Insulatard and Actrapid in combination (n = 1). Six of the T2DSI subjects received oral anti-diabetic treatment (Metformin only). The T2DOAD group (n = 7) was treated with either diet alone (n = 2), or diet combined with metformin (n = 3), or sulfonylurea and rosiglitazone (n = 2). Additional medication among the three groups comprised the following: in the T2DSI group: statins (n = 7), antihypertensive treatment (n = 7), acetylsalicylic acid (n = 7), levothyroxine (n = 1), antidepressants (n = 2), antacida (n = 1), NSAID besides acetylsalicylic acid (n = 1); in the T2DOAD group: statins (n = 5), antihypertensive treatment (n = 7), acetylsalicylic acid (n = 2), levothyroxine (n = 2), allopurinol (n = 2), NSAID besides acetylsalicylic acid (n = 1) and in the Control group: NSAID besides acetylsalicylic acid (n = 1) and fenytoin (n = 1).

**Table 1:** Clinical and metabolic characteristics.

	Controls	T2DOAD	T2DSI	P-value
Men/Women	6/1	3/4	5/2	
Insulin requirements (IU/day)			195±26	
Age (Years)	59.4±2.0	64.0±3.2	58.9±2.6	0.34
BMI (kg/m^2^)	28.0±1.5	31.8±2.4	35.7±2.1	0.050
Fasting plasma glucose (mmol/l)	5.3±0.2 ^a^	7.8±0.3 b	7.9±0.4	<0.001
HbA_1c_ (%)		6.8±0.2	8.8±0.6	0.006^e^
Insulin (pmol/l)	68±8 ^a^	126±32^a^	350±46	<0.001
C-peptide (pmol/l)	809±100	1568±241^ac^	641±148	0.003
Total cholesterol (mmol/l)	5.7±0.3 ^a^	4.3±0.2^b^	4.0±0.3	<0.001
LDL cholesterol (mmol/l)	3.5 (3.3; 3.6)	2.0 (1.8; 2.0)^d^	2.2 (1.7; 2.4)^d^	0.002
HDL cholesterol (mmol/l)	1.6 (1.2; 1.9)	1.4 (1.0; 1.8)	1.0 (0.8; 1.2) (n = 6)^c^	0.04
Triglycerides (mmol/l)	1.4 (1.2; 1.6)	1.5 (0.9; 3.3)	2.6 (1.4; 3.0)	0.63
Systolic BP (mmHg)	129±5	136±4	140±5	0.21
Diastolic BP (mmHg)	85±2	75±3	82±4	0.09
Serum creatinine (μmol/l)	77±5	71±5	85±7	0.25
Activity (hours/week)	2.3 (0.0; 4.0)	3.0 (1.9; 6.3)	0.0 (0.0; 1.8)	0.06
Diabetes duration (Years)		3.9±1.5	17.3±4.1	0.01

All data are presented as means±SE or median (range), (n  = 7 unless indicated otherwise). T2DSI  =  type 2 diabetes, severely insulin resistant, T2DOAD  =  type 2 diabetes, oral antidiabetics, diet.

M-value, Insulin and C-peptide are mean values obtained during the last 30 minutes of the hyperinsulinemic euglycemic clamp.

P-value is result of ANOVA testing. Post-hoc test were performed when indicated:

a. P<0.001 vs. T2DSI,

b P<0.001 vs. controls,

c P≤0.01 vs. controls,

d P<0.05 vs. controls.

BMI did not differ significantly among the groups, although there was a tendency for increased BMI in the TD2SI group (P = 0.05). Body composition, expressed by TBW, was 25.3±1.3, 26.2±1.7 and 29.6±0.9 in controls, T2DOAD, and T2SI respectively and were not statistically significantly different (P = 0.15). Diabetes duration was ∼4 fold longer and HbA_1c_ was significantly higher among T2DSI compared to T2DOAD. The M-value, a measurement of insulin sensitivity, was ∼50% lower in the T2DSI group compared to the T2DOAD group (Table 2). All groups were characterized by a low physical activity level (<3 hours weekly) and none of the participants participated in any kind of vigorous exercise. Total and LDL cholesterol were significantly higher among controls compared to the diabetic groups.

**Table 2:** Comparison of clamp data (last 30 minutes of the hyperinsulinemic euglycemic clamp).

	T2DOAD	T2DSI	P-value
M-value (mg/kg/min)	2.9(2.4; 5.5)	1.3(1.0; 2.9)	0.037
Insulin (pmol/l)	796±93	1112±62	0.016
C-peptide (pmol/l)	1126±218	364±96	0.017

All data are presented as means±SE or median (range) (n = 7). T2DSI  =  type 2 diabetes, severely insulin resistant, T2DOAD  =  type 2 diabetes, oral antidiabetics, diet.

**Figure 1:**
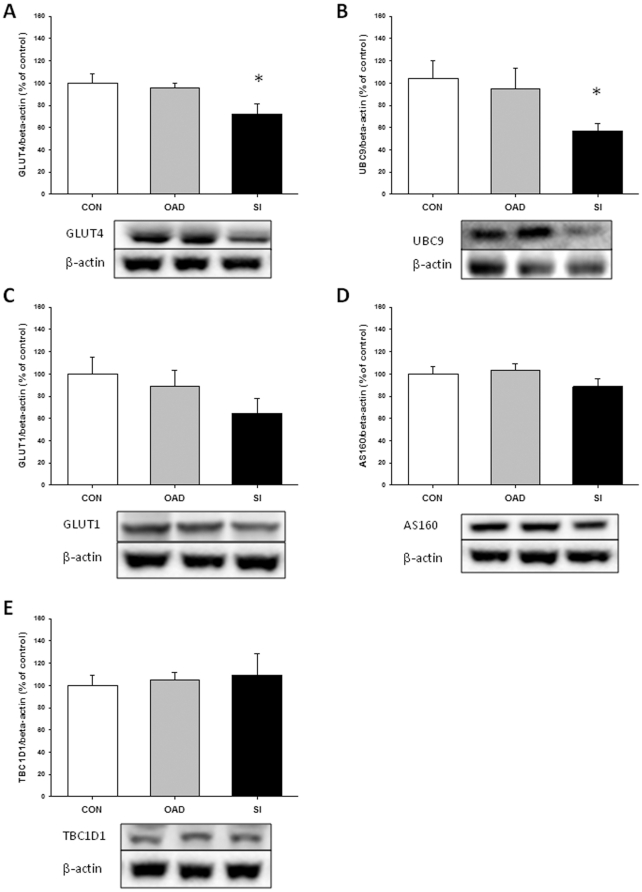
Total protein content in muscle biopsies. Protein content was assessed by western blotting analysis of GLUT4 (A), UBC9 (B), GLUT1 (C), TBC1D1 (D), and AS160 (E). All protein expressions are corrected for β-actin levels. Data are presented as mean + SE. *: P<0.05 versus control and T2DOAD.

**Figure 2:**
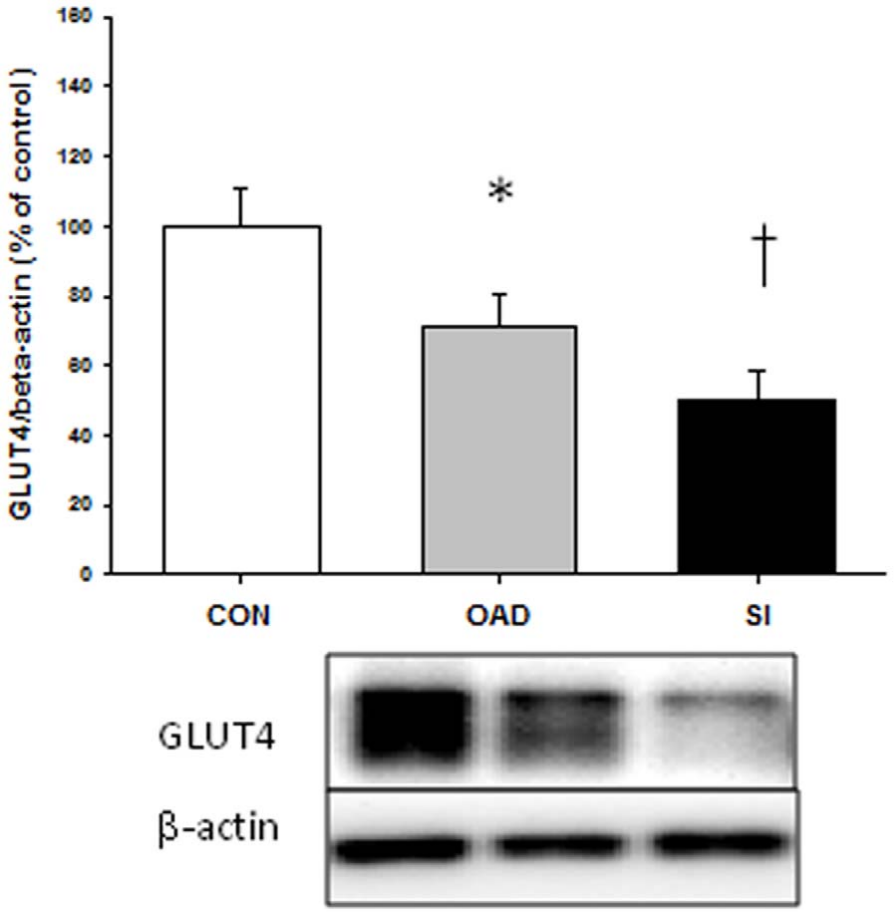
GLUT4 protein content in fat biopsies. Protein content was assessed by western blotting analysis of GLUT4 and corrected for β-actin levels. Data are presented as mean + SE. *: P<0.05 versus control and T2DOAD.

**Figure 3:**
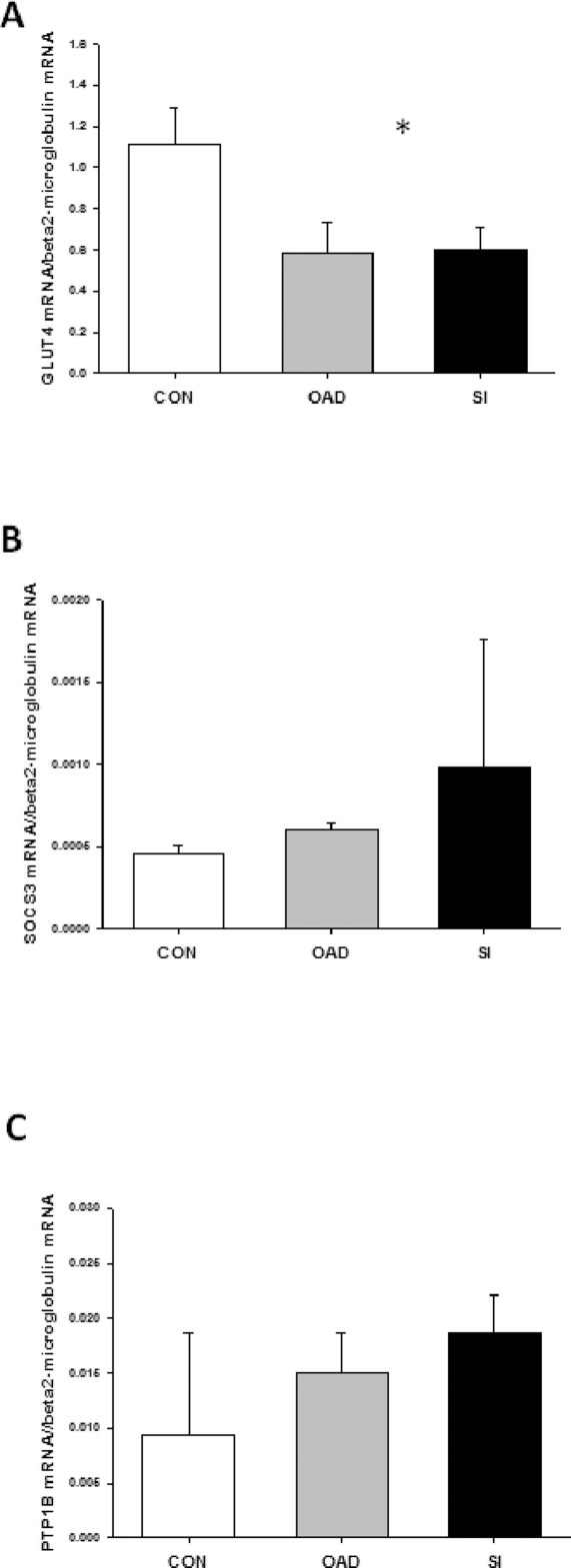
Gene expression of GLUT4, SOCS3 and PTP1B. mRNA levels in skeletal muscle was measured by quantitative PCR using beta2-microglobulin as housekeeping gene. GLUT4 mRNA was reduced in skeletal muscle from both diabetic groups. GLUT4 gene expression is presented as mean + SE. SOCS3 and PTP1B expressions were not normally distributed and data are presented as median + 75% percentile *: P<0.05 versus control and T2DOAD.

As depicted in Figure 1A GLUT4 expression, normalized for β-actin, was reduced by ∼30% in skeletal muscle from the T2DSI group with no difference between controls and type 2 diabetic patients in the T2DOAD group. This was associated with a decreased expression of UBC9 (Fig 1B). GLUT1 expression was also reduced in skeletal muscle in the T2DSI group, but this difference did not reach statistical significance (P = 0.21). Expression of TBC1D1 and AS160 in skeletal muscle was similar among type 2 diabetic patients and matched controls. As shown in figure 2, GLUT4 expression in adipose tissue was reduced in both groups of diabetic patients with the most predominant reduction in patients with severe insulin resistance. GLUT4 gene expression in skeletal muscle was decreased in both diabetic groups as shown on figure 3A. We could only detect low gene expression levels of SOCS3 and PTP1B (Fig 3B and 3C), both negative regulators of insulin signaling [17], [18], and the differences in expression levels were not statistically significant.

## Discussion

The data from the current study demonstrate that reduced GLUT4 expression in skeletal muscle is a pathological component in a subgroup of type 2 diabetic patients with severe insulin resistance. This finding challenges the current dogma, that GLUT4 expression is normal in muscle from type 2 diabetic patients [2], [9]–[11]. This perception is mainly based on data from patients that do not require insulin, and in agreement with these findings we did not see reduced GLUT4 expression in a matched group of patients not treated with insulin. Our observation was done in a small group of subject and larger studies are needed to determine to which degree reduced GLUT4 expression is a part of the pathogenesis behind insulin resistance in other groups of type 2 diabetic patients.

GLUT4 protein levels are positively associated with insulin sensitivity in humans [1], [19], but GLUT4 expression does not necessarily reflect translocation to the cell surface during insulin stimulation. SOCS3 and PTP1B levels were not significantly increased in the diabetic subjects, but it is well-established that patients with type 2 diabetes mellitus have defects in the insulin signaling cascade that stimulate GLUT4 translocation [2]. Therefore the combination of impaired insulin signaling and reduced GLUT4 expression is likely to further aggravate the therapeutic challenge of normalizing glucose levels among this group of diabetic patients.

Gene expression of GLUT4 was reduced in both groups of diabetic patients, but only patients with severe insulin resistance had decreased UBC9 expression. This indicates that the reduced GLUT4 protein level is due to a combined effect of reduced production and increased GLUT4 degradation. In healthy humans, prolonged insulin stimulation during a hyperinsulinemic euglycemic clamp is associated with a reduction of GLUT4 protein levels in skeletal muscle [20]. It is possible that prolonged stimulation with exogenous insulin disrupts GLUT4 recycling, and this leads to increased GLUT4 degradation. In patients with severe insulin resistance this could cause a vicious cycle where the progressive increase in insulin dosage cause a reduction in GLUT4 levels and thereby increase the need for further insulin treatment.

Reduced GLUT4 levels in adipose tissue from patients with type 2 diabetes have previously been observed [2]. This defect is also present in adipose tissue from subject in high risk of developing diabetes [21], [22] and is associated with alterations in the endocrine function of adipocytes [23]. Our finding show that GLUT4 expression in further decreased in patients with severe insulin resistance compared to diabetic patients with a less severe insulin resistance.

Skeletal muscle also expresses the glucose transporter GLUT1, although at lower levels than GLUT4 [2]. GLUT1 is mainly responsible for basal glucose uptake, but an increase in GLUT1 expression could potentially compensate for a reduced GLUT4 level. However, similar to previous observations [11] we saw a tendency to lower GLUT1 expression in patients with severe insulin resistance, although this difference did not reach statistical significance. TBC1D1 and AS160 are negative regulators of insulin stimulated glucose uptake [3], but the severe insulin resistance was not associated with alterations of TBC1D1 and AS160 on a protein expression level.

In conclusion, we found that GLUT4 expression is reduced in skeletal muscle from type 2 diabetic patients who despite being treated with more than 100 units of insulin daily have poor glycemic control. This challenges the dogma that altered GLUT4 protein levels do not contribute to insulin resistance in humans. Instead, reduced GLUT4 expression may partly explain the severe insulin resistance and poor glycemic control among type 2 diabetic patients with severe insulin resistance.
